# Lsd1 Restricts the Number of Germline Stem Cells by Regulating Multiple Targets in Escort Cells

**DOI:** 10.1371/journal.pgen.1004200

**Published:** 2014-03-13

**Authors:** Susan Eliazer, Victor Palacios, Zhaohui Wang, Rahul K. Kollipara, Ralf Kittler, Michael Buszczak

**Affiliations:** 1 Department of Molecular Biology, University of Texas Southwestern Medical Center at Dallas, Dallas, Texas, United States of America; 2 McDermott Center for Human Growth and Development, University of Texas Southwestern Medical Center at Dallas, Dallas, Texas, United States of America; 3 Department of Pharmacology, University of Texas Southwestern Medical Center at Dallas, Dallas, Texas, United States of America; Harvard Medical School, Howard Hughes Medical Institute, United States of America

## Abstract

Specialized microenvironments called niches regulate tissue homeostasis by controlling the balance between stem cell self-renewal and the differentiation of stem cell daughters. However the mechanisms that govern the formation, size and signaling of *in vivo* niches remain poorly understood. Loss of the highly conserved histone demethylase *Lsd1* in *Drosophila* escort cells results in increased BMP signaling outside the cap cell niche and an expanded germline stem cell (GSC) phenotype. Here we present evidence that loss of *Lsd1* also results in gradual changes in escort cell morphology and their eventual death. To better characterize the function of Lsd1 in different cell populations within the ovary, we performed Chromatin immunoprecipitation coupled with massive parallel sequencing (ChIP-seq). This analysis shows that Lsd1 associates with a surprisingly limited number of sites in escort cells and fewer, and often, different sites in cap cells. These findings indicate that Lsd1 exhibits highly selective binding that depends greatly on specific cellular contexts. Lsd1 does not directly target the *dpp* locus in escort cells. Instead, Lsd1 regulates *engrailed* expression and disruption of *engrailed* and its putative downstream target *hedgehog* suppress the *Lsd1* mutant phenotype. Interestingly, over-expression of *engrailed*, but not *hedgehog*, results in an expansion of GSC cells, marked by the expansion of BMP signaling. Knockdown of other potential direct Lsd1 target genes, not obviously linked to BMP signaling, also partially suppresses the *Lsd1* mutant phenotype. These results suggest that Lsd1 restricts the number of GSC-like cells by regulating a diverse group of genes and provide further evidence that escort cell function must be carefully controlled during development and adulthood to ensure proper germline differentiation.

## Author Summary

The mechanisms that govern the formation, size and signaling output of *in vivo* niches remain poorly understood. Studies of *Drosophila* germline stem cells (GSCs) have suggested that chromatin programming greatly influences the behavior of these cells and their progeny. Previous work has shown that loss of the highly conserved histone demethylase Lsd1 results in ectopic niche signaling and an expanded GSC phenotype. To determine direct regulatory targets of Lsd1, we employed chromatin immunoprecipitation coupled with massive parallel sequencing (ChIP-seq) using specific cell populations inside and outside of the GSC niche. These experiments revealed that Lsd1 exhibits highly enriched binding to over one hundred genomic sites within a specific cell population. Furthermore, mis-regulation of some of these direct targets contributes to the expanded stem cell phenotype observed in *Lsd1* mutants. These results provide insights into how Lsd1 directly restricts the size of the GSC microenvironment and establish a platform for understanding and exploring chromatin programming inside and outside an *in vivo* stem cell niche.

## Introduction

Stem cells undergo self-renewing divisions in which at least one daughter retains its stem cell identity, while the second daughter may or may not differentiate, depending on intrinsic and extrinsic cues. A balance between stem cell self-renewal and differentiation must be maintained for proper organ formation during development and tissue homeostasis in adulthood. Stem cells often reside in microenvironments called niches, and specific mechanisms tightly regulate the size and signaling output of these structures [1]. However, *in vivo* niches have often proven difficult to identify in mammalian tissues. As a result, much of the current understanding of niches stems from the study of invertebrate models such as the germline stem cells (GSCs) of the *Drosophila* ovary.

**Figure 1:**
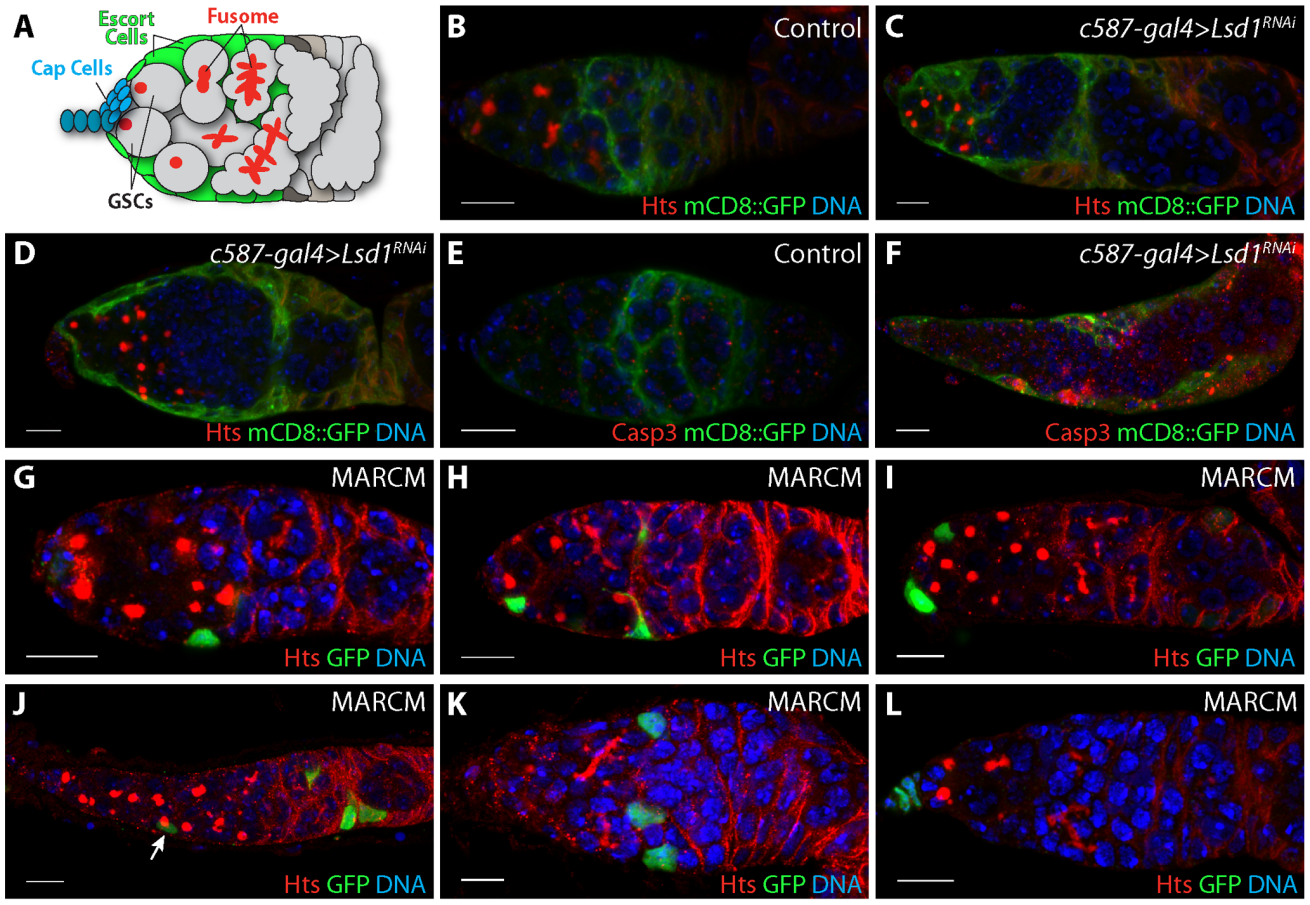
Further characterization of the *Lsd1* mutant phenotype. (A) Schematic of a *Drosophila* germarium. Two germline stem cells (GSCs) reside next to terminal filament and cap cells labeled in blue. GSCs carry a round endoplasmic reticulum-like organelle called a fusome or spectrosome. These fusomes become branched in appearance as GSC daughters form multicellular cysts. Lining the anterior end of the germarium next to the cap cells are the escort cells labeled in green. Germaria from (B) *c587-gal4>mCD8::GFP* and (C,D) *c587-gal4>mCD8::GFP+Lsd1^RNAi^* three day old females stained for Hts (red), GFP (green) and DNA (blue). RNAi knockdown of *Lsd1* results in the accumulation single cells with round fusomes and the gradual retraction of escort cell extensions. (E) *c587-gal4>mCD8::GFP* and (F) *c587-gal4>mCD8::GFP+Lsd1^RNAi^* germaria from females shifted to 29°C for 3 days stained for activated Caspase 3 (Casp3), GFP (green) and DNA (blue). (G–L) MARCM *Lsd1^ΔN^* clones stained for Hts (red), GFP (green) and DNA (blue). *Lsd1* mutant escort cell clones are associated with undifferentiated germ cells with round fusomes. Scale bars = 10 µM.

*Drosophila* female GSCs reside in a well-characterized niche at the tip of a structure called a germarium (Figure 1A). Within germaria, GSCs lie immediately next to a somatic cell niche comprised of cap cells and terminal filament cells [2]. Escort cells reside adjacent to the cap cells and line the anterior portion of the germarium. These cells act to shepherd the germ cells during the earliest stages of their differentiation [3], [4], after which developing germline cysts are enveloped by follicle cells derived from a second stem cell population within the germarium. Cap cells produce Decapentaplegic (Dpp), which in turn activates a canonical Bone Morphogenic Protein (BMP) signal transduction pathway in GSCs [5], [6]. BMP pathway activation results in the transcriptional repression of *bag of marbles* (*bam*) [7]–[9], a factor both necessary and sufficient for germ cell differentiation [10], [11]. Ectopic Dpp signaling outside the tip of the germarium results in an expanded GSC phenotype [5], [9].

Other pathways and neighboring cells likely regulate niche specific BMP signaling. For example, a recent study provides evidence that *hedgehog* (*hh*) produced by the cap cells stimulates the anterior escort cells to produce niche specific signals [12] Moreover, several additional intrinsic and extrinsic mechanisms help restrict Dpp ligand production and diffusion within the niche (reviewed in [13], [14]). One such mechanism involves the histone demethylase Lysine Specific Demethylase 1 (Lsd1). Lsd1 uses a flavin-dependent monoamine oxidative based mechanism to remove mono- and di-methyl groups from histone H3 on lysine 4 (H3K4me1 and H3K4me2) [15]. In mammals, Lsd1 has been shown to silence a number of distinct gene sets in different cellular contexts, including Notch targets, TGFβ-1 and various loci involved in the maintenance of embryonic stem cells [16]–[20]. Additional studies suggest that Lsd1 may also promote gene expression under certain circumstances [21]. Disruption of *Drosophila Lsd1* results in a male and female sterile phenotype, marked by the expansion of GSC-like cells in the germarium [22], [23]. These cells exhibit ectopic BMP responsiveness and fail to initiate a normal differentiation program once they leave the cap cell niche [24].

To characterize the molecular mechanism by which Lsd1 restricts signaling outside the *Drosophila* female GSC niche, we used ChIP-seq to define direct binding sites of Lsd1 specifically in either escort cells or cap cells. These experiments revealed that Lsd1 binds to over one hundred sites in escort cells and provide further insights into how Lsd1 contributes to the chromatin programming of cells inside and outside of an *in vivo* niche.

## Results

### Further characterization of the *Lsd1* loss-of-function phenotype

Escort cells send out extensions that closely contact germline cysts undergoing the early steps of differentiation [3], [4]. Escort cell death or genetically disrupting escort cell extensions can lead to an inappropriate expansion of GSC-like cells in the germarium [3]. Previous results showed that Lsd1 functions within escort cells to prevent expanded BMP signaling outside of the GSC niche [24]. This phenotype was accompanied by widespread cell death in both somatic cells and germ cells. Therefore we considered the possibility that the expansion of BMP signaling exhibited by *Lsd1* mutants may depend on changes to escort cell morphology and number. To test this, we knocked down the expression of *Lsd1* specifically in the escort cells and early follicle cells by crossing *UAS-Lsd1^RNAi^* into a *c587-gal4; UAS-mCD8::GFP* background and stained the resulting ovaries for GFP and the fusome marker Hts. Fusomes are highly vesiculated organelles that appear round in GSCs and cystoblasts, and become branched as these germ cells differentiate into multi-cellular cysts [25], [26]. Three days after eclosion, control samples appeared normal. These germaria typically contained two to four single cells (GSCs and cystoblasts) with round fusomes and escort cells that extended cytoplasmic processes between the developing cysts (Figure 1B). In contrast, the *Lsd1* RNAi samples showed an expansion of GSC-like cells with round fusomes. Escort cell extensions were clearly present in some germaria, but were missing in others (Figure 1C,D). These observations suggested that while the knockdown of *Lsd1* caused changes in escort cell morphology, the presence of extra single cells with round fusomes did not absolutely depend on a complete loss of escort cell extensions. However changes in escort cell morphology likely contributed to the phenotype over time. In addition, expression of *Lsd1^RNAi^* also led to an increase of death within escort cells, consistent with the widespread cell death previously noted in *Lsd1* null mutant germaria (Figure 1E,F) [24].

Next we performed clonal analysis using the mosaic analysis with a repressible cell marker (MARCM) system to further analyze the *Lsd1* mutant phenotype. Clonal germaria were stained for the positive clone marker GFP and for the fusome marker Hts. We categorized the relative position of escort cell clones along the anterior-posterior axis of the germarium. Cells were considered anterior escort cells if they were immediately next to the cap cells, posterior escort cells if they were immediately adjacent to the follicle stem cells and middle escort cells if they were located in any position in between. We induced control and *Lsd1* mutant clones in parallel. Control escort cell clones were never associated with an obvious robust germline tumor phenotype, although we noted one exception in which a single germarium with control escort cell clones contained six single germline cells with round fusomes (1 out of 143 counted). By contrast, 17% (27/155) of the germaria that contained *Lsd1* mutant escort cell clones displayed an expanded germline stem cell-like cell phenotype (Figure 1G–J). The relative position of *Lsd1* mutant clones appeared to correlate with the appearance of a germline phenotype. The vast majority of germaria (96%; n = 27) that contained greater than 5 germline stem cell-like cells carried at least one *Lsd1* mutant middle escort cell clone. We observed one example in which a germarium with a mild expansion of GSC-like cells contained an *Lsd1* mutant anterior clone and posterior clone but no middle escort cell clones (Figure 1H). Of note, most germaria that carried middle escort cell clones did not exhibit a GSC expansion phenotype (98/125 germaria). While their appearance was rare, germaria with only anteriorly or posteriorly (Figure 1K) positioned escort cell clones did not display a robust GSC-like cell expansion phenotype. Similarly, loss of *Lsd1* in the terminal filament did not result in an obvious phenotype (Figure 1L).

Germaria that contained *Lsd1* mutant escort cell clones and exhibited an increased number of GSCs occasionally had an elongated and abnormal morphology (Figure 1J). Moreover, 22.1% of the germaria (n = 199) from *Lsd1* mutant females lacked marked escort cell clones, compared to 13.9% of control germaria (n = 166), and the average number of *Lsd1* mutant clones per germarium (4.72 escort cell clones/germarium) was lower compared to controls (8.77 escort cell clones/germarium), suggesting that either *Lsd1* mutant escort cell progenitors exhibited reduced proliferation during development or died during the course of the experiment. If increased death within the escort cell population accounted for all the observed phenotypes, one might predict that complete loss of all *Lsd1* mutant escort cell clones within a particular germarium would result in an increased number of GSC-like cells. However, we did not observe an expanded GSC phenotype in germaria that lacked clones from *Lsd1* mutant females. Together all the phenotypic data suggest both that escort cells require *Lsd1* function to limit GSC number and that loss of *Lsd1* compromises the growth and survival of escort cells, consistent with previous observations [24] and those noted above, which in turn further exacerbates the observed germ cell phenotypes.

### Mapping Lsd1 binding sites in specific cell populations within the germarium

**Figure 2:**
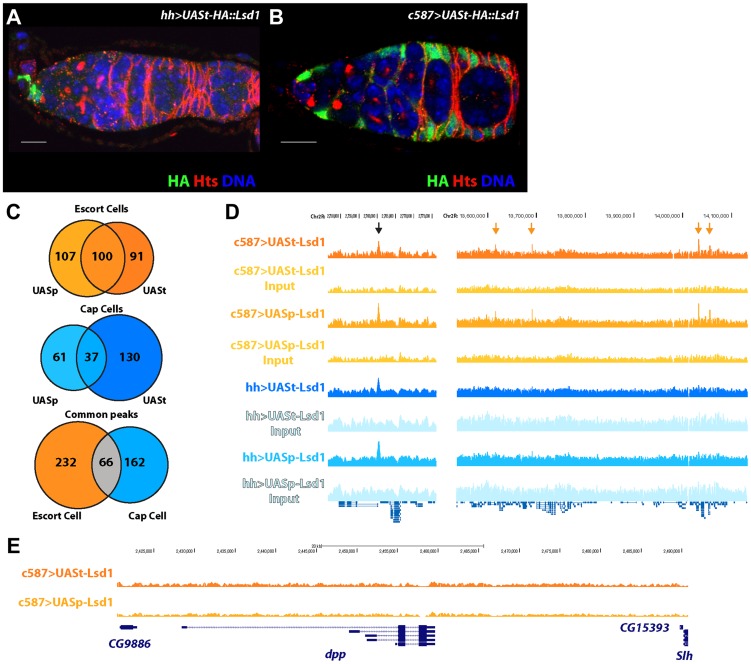
Defining the targets of Lsd1 in the cap cell niche and the surrounding escort cells within *Drosophila* germaria. (A,B) Germaria stained for HA (green), to label the transgene, Hts (red), to label fusomes, and DNA (blue) (Scale bars, 10 µM). (A) *hh-gal4>UASt-HA::Lsd1* germarium showing expression in cap cells and (B) *c587-gal4>UASt-HA::Lsd1* germarium displaying expression in the escort cells and early follicle cells, but not in the cap cells. (C) Venn diagram showing the number of Lsd1 binding sites in escort cells and cap cells. (D) Lsd1 binding peaks on a segment of Chr2R. Black arrow points to a common peak in both the escort cell and cap cell populations. Orange arrows point to unique peaks seen only in the escort cell and early follicle cell population. (E) No Lsd1 binding sites are observed around the *dpp* locus in escort cells.

To directly define the molecular mechanisms by which Lsd1 influences escort cell function, we elected to identify direct targets of Lsd1 regulation in these cells. Determining whether Lsd1 targeted potential candidate genes represented a significant challenge. For example, the size and complexity of the *dpp* promoter precluded our ability to assay whether Lsd1 directly targeted this gene using a PCR based chromatin immunoprecipitation (ChIP) approach. To systematically define Lsd1 binding sites, we conducted ChIP experiments coupled with massive parallel sequencing (ChIP-seq). We used a number of different Hemagglutinin (HA) tagged transgenes, including a N-terminally tagged *UASt-HA::Lsd1* transgene that exhibits high expression in the somatic cells and a N-terminally tagged *UASp-HA::Lsd1* transgene that displays relatively lower levels of expression in the somatic cells (Figure 2; S1). The *UASt-HA::Lsd1* and *UASp-HA::Lsd1* transgenes both fully rescued the *Lsd1^ΔN^* GSC tumor phenotype when driven by the *c587-gal4* driver. Because Lsd1 is expressed ubiquitously throughout the ovary [24], we sought to determine whether this protein bound to distinct sites in different cell populations within the germarium. We expressed the *UASt-HA::Lsd1* and *UASp-HA::Lsd1* trangenes in cap cells and terminal filament cells using *hh-gal4* (Figure 2A; S1) and in the escort cells and early follicle cells using the *c587-gal4* driver (Figure 2B; S1). HA-directed ChIP assays were performed on dissected ovaries and the immunoprecipitated chromatin was compared to input chromatin as a control. Within escort cells and early follicle cells, products of the *UASp-HA::Lsd1* and *UASt-HA::Lsd1* trangenes bound to 207 and 191 sites respectively (Based on the FindPeaks algorithm using a p-value threshold of 1.00e-3 to maximize the number of potential peaks; Table S1, S2), with 100 common sites sharing some degree of overlap (Figure 2C). Within cap cells and terminal filament cells, the *UASp-HA::Lsd1* and *UASt-HA::Lsd1* transgenic products associated with 98 and 167 genomic loci respectively (Table S3, S4), with 37 overlapping loci in common between the two datasets (Figure 2C). Comparing all four datasets revealed 66 common peaks between terminal filament/cap cells and escort cells/early follicle cells (Figure 2C,D). 232 peaks appeared specific for escort cells and early follicle cells and 162 specific for cap cells and terminal filament cells (Figure 2C,D). MACs analysis [27] showed similar but broader peak calls (Table S5, S6, S7, S8). Lsd1 enrichment peaks were spread throughout the *Drosophila* genome (Figure S2) and showed a preference for the promoter and 5′UTR regions of genes (Figure S3). We were unable to isolate a sufficient number of cells to map H3K4 methylation across the escort cell genome. However, comparing our data with available data from the modENCODE project revealed that Lsd1 binding peaks correlate with valleys of H3K4 methylation in embryos (Figure S4), consistent with the established biochemical activity of Lsd1.

Strikingly, we did not observe any enrichment for Lsd1 binding near the *dpp* locus in escort cells (Figure 2E), indicating that the repression of BMP signal transduction by Lsd1 must be through an indirect mechanism. We examined the annotation of genes near escort cell and early follicle cell peaks, cap cell and terminal filament peaks, shared peaks and from the UASt-HA::Lsd1 data sets (Table S9, S10, S11, S12). This analysis indicated that genes near escort cell specific Lsd1 binding peaks encode products with a diverse array of functions needed for development, basic cellular processes and reproduction (Figure S5A) [28], [29]. Further analysis of this gene set did not reveal significant enrichment for components of specific pathways. MEME analysis showed an enrichment of ACTGGAA elements within Lsd1 binding sites (Figure S5B). The significance of this enrichment remains unclear. These results suggested that the mis-expression of a functionally diverse set of genes likely contributes to the *Lsd1* mutant phenotypes.

### *engrailed* and *hedgehog* mis-regulation contribute to the *Lsd1* mutant phenotype

**Figure 3:**
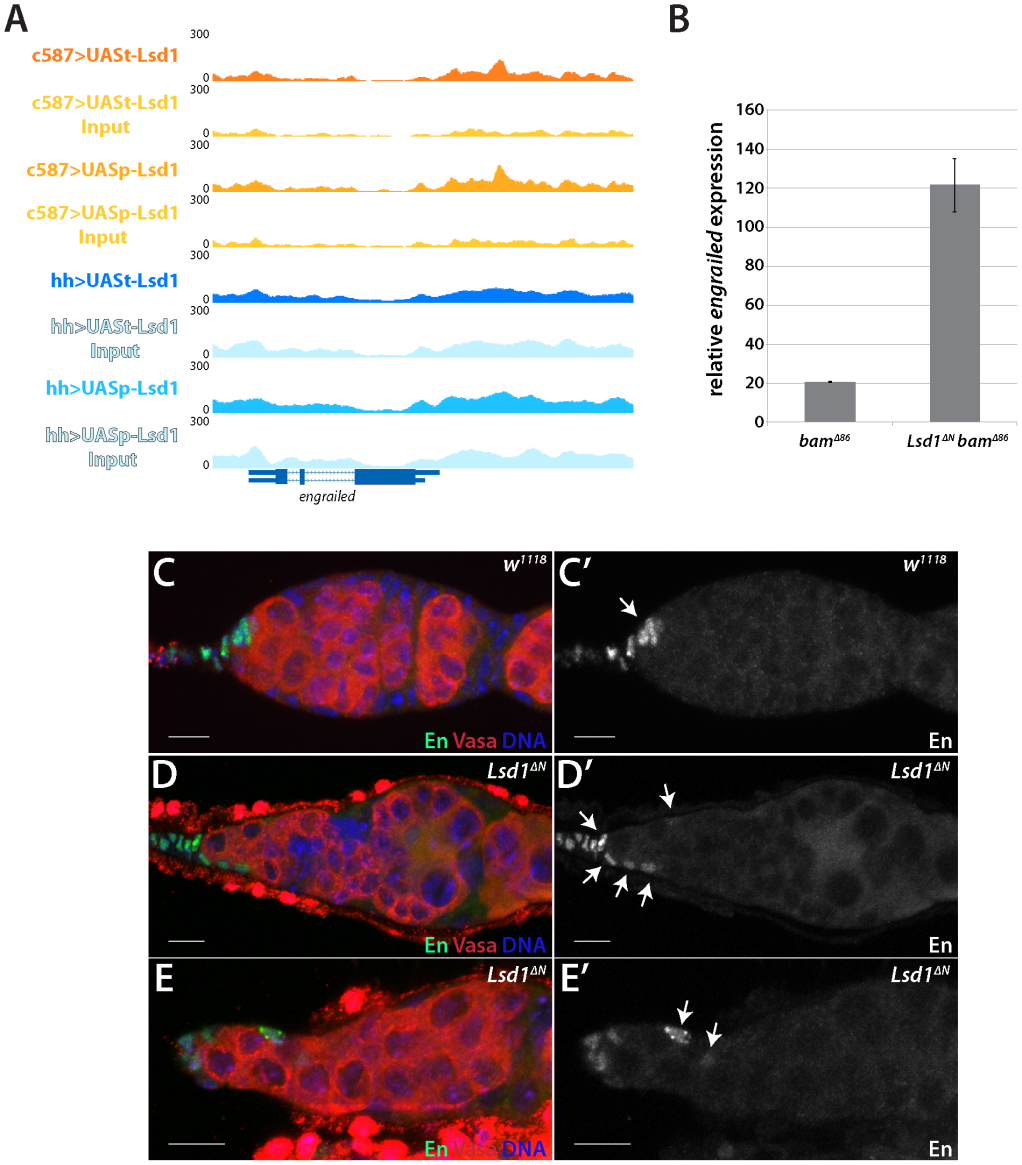
Lsd1 silences *engrailed* in the escort cells. (A) A Lsd1 binding peak is observed in the *engrailed* promoter in escort cells. (B) RT-qPCR of whole ovary extracts from *bam^Δ86^* and *bam^Δ86^ Lsd1^ΔN^* double mutants. The *bam^Δ86^ Lsd1^ΔN^* double mutant samples exhibit a relative 6-fold increase of *engrailed* transcripts when compared to *bam^Δ86^* mutants. (C and C′) *w^1118^* and (D–E′) *Lsd1^ΔN^* homozygous germaria stained for Engrailed (En) (green) and VASA (red) and DNA (blue). Arrows point to Engrailed positive cells. In *w^1118^* control germaria, Engrailed is expressed in the terminal filament and cap cells, whereas *Lsd1* mutants display engrailed expression in the anterior escort cells. Scale bars = 10 µM.

The *engrailed* gene stood out as one potentially relevant target among the list of candidate genes. *engrailed* encodes a homeobox transcription factor that acts as a segment polarity gene during embryogenesis [30]–[32]. Previous results showed that *engrailed* regulates early ovarian development and that Engrailed protein expression is restricted to the terminal filament and cap cells in adult germaria [33]. Engrailed functions within these cells to help maintain GSCs [12]. Our ChIP-seq data revealed that Lsd1 exhibits enriched binding to a 2 kb region of the *engrailed* promoter in the escort cells (Figure 3A). We performed RNA RT-qPCR to look at the transcript levels of *engrailed* in *Lsd1* mutants. We compared *bam* mutants to *bam Lsd1* double mutants because these samples are comparable in size and have the same basic cellular makeup (Figure S6). This analysis revealed that *engrailed* transcript levels increased 6 fold in the absence of *Lsd1* (Figure 3B).

Next, we tested whether Engrailed protein expression expanded in the absence of *Lsd1*. In wild type germaria, cap cells and terminal filament cells express readily detectable levels of Engrailed, whereas the escort cells do not (Figure 3C) [12], [33]. In *Lsd1^ΔN^* mutants, however, we observed ectopic Engrailed protein expression in a limited number of escort cells, in addition to the terminal filament and cap cells, in 85.7% (n = 91) of the germaria examined (Figure 3D). These Engrailed expressing escort cells tended to be positioned immediately adjacent to the normal niche, although occasionally we observed Engrailed expressing escort cells several cell positions away from the cap cells (Figure 3E). We cannot rule out the possibility that other cells also mis-expressed Engrailed, but at a level below our detection threshold. These data together suggest that Lsd1 serves to repress *engrailed* expression within a subpopulation of escort cells.

**Figure 4:**
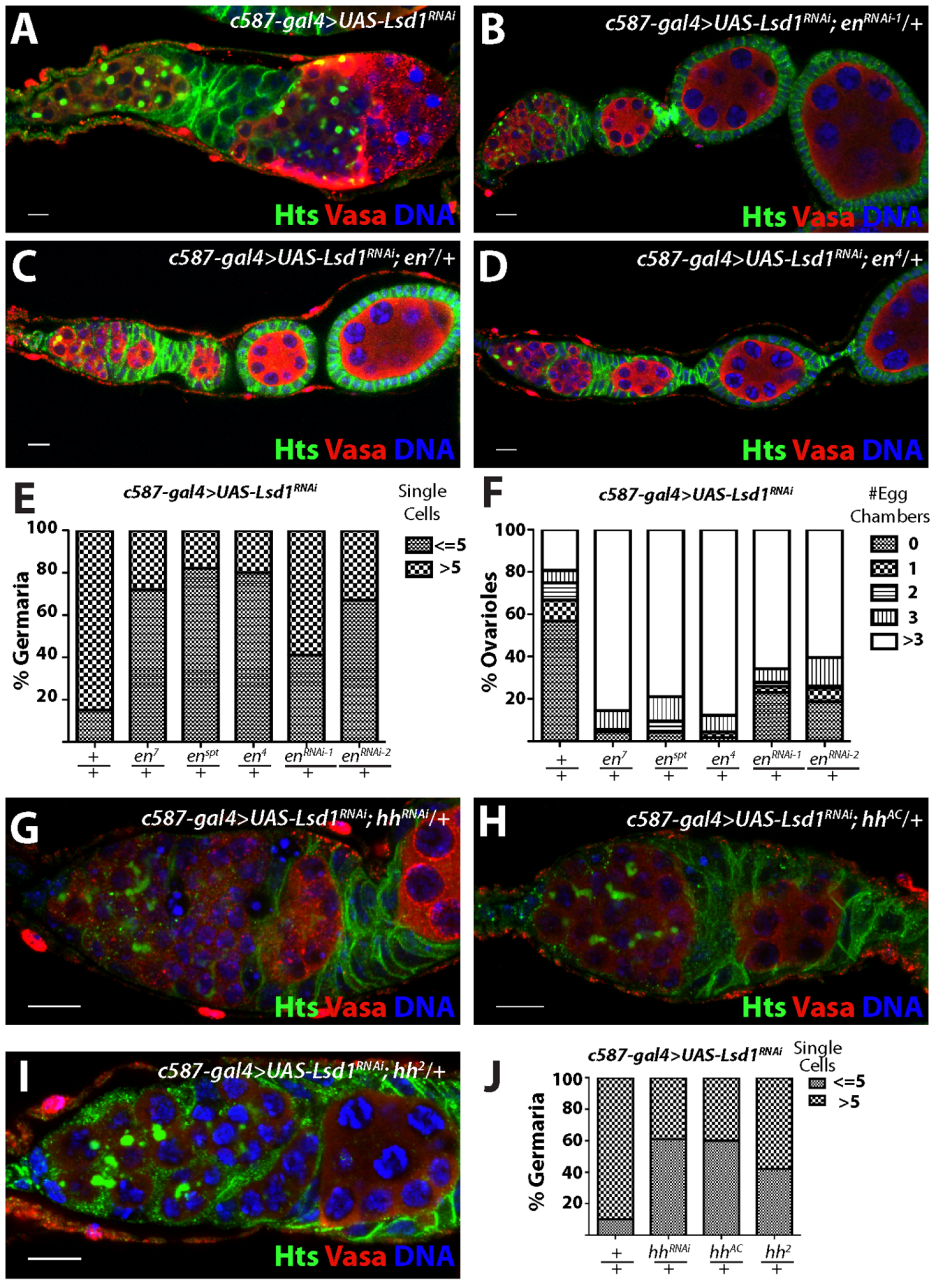
*engrailed* mutations suppress the *Lsd1* RNAi phenotype. Ovarioles from (A) *c587-gal4*>*UAS-Lsd1^RNAi^* (B) *c587-gal4*>*UAS-Lsd1^RNAi^; UAS en* ^RNAi-1^ */+* (C) *c587-gal4*>*UAS-Lsd1^RNAi^; en^7^/+* and (D) *c587-gal4*>*UAS-Lsd1^RNAi^; en^4^/+* stained for Hts (green), VASA (red) and DNA (blue). (E) Graph showing the percentage of germaria that contain the indicated number of single germ cells with round fusomes for each genotype. (F) Graph showing the percentage of ovarioles that have a given number of developing egg chambers for each genotype. c*587-gal4*>*UAS-Lsd1^RNAi^* (n = 171); c*587-gal4*>*UAS-Lsd1^RNAi^*; *en^7^/+* (n = 90); c*587-gal4*>*UAS-Lsd1^RNAi^*; *en^spt^/+* (n = 95); c*587-gal4*>*UAS-Lsd1^RNAi^*; *en^4^/+* (n = 115); c*587-gal4*>*UAS-Lsd1^RNAi^*; *en^RNAi-1^/+* (n = 108); c*587-gal4*>*UAS-Lsd1^RNAi^*; *en^RNAi-2^/+* (n = 96). (G) *c587-gal4*>*UAS-Lsd1^RNAi^; UAS hh* ^RNAi^ */+* (H) *c587-gal4*>*UAS-Lsd1^RNAi^; hh^AC^/+* and (I) *c587-gal4*>*UAS-Lsd1^RNAi^; hh^2^/+* stained for Hts (green), VASA (red) and DNA (blue). (J) Graph showing the percentage of germaria that contain the indicated number of single germ cells with round fusomes for each genotype. c*587-gal4*>*UAS-Lsd1^RNAi^* (n = 307); *c587-gal4*>*UAS-Lsd1^RNAi^; UAS hh* ^RNAi^ */+* (n = 222) *c587-gal4*>*UAS-Lsd1^RNAi^; hh^AC^/+* (n = 88) and *c587-gal4*>*UAS-Lsd1^RNAi^; hh^2^/+* (n = 184). Scale bars = 10 µM.

To test the functional relevance of ectopic Engrailed expression in escort cells, we assayed whether disruption of *engrailed* function, either through RNAi knockdown or loss-of-function mutations, modified the *Lsd1* mutant phenotype. Knockdown of *engrailed* in an *Lsd1* RNAi background (Figure 4A) suppressed the expanded GSC-like cell *Lsd1* mutant phenotype (Figure 4B,E,F). Furthermore, three independent *engrailed* mutations also suppressed the *Lsd1* RNAi-induced phenotype, so that the number of single cells with round fusomes decreased and cyst development within the germarium proceeded normally (Figure 4C–F). In all cases, *engrailed* suppression of the *Lsd1* RNAi phenotype resulted in the formation of morphologically normal ovarioles with maturing egg chambers.

In *Drosophila*, Engrailed drives the expression of *hedgehog* (*hh*), which in turn leads to the expression of *dpp* in adjacent cells [34]–[37]. Previous analysis showed an expansion of *hh* expression in *Lsd1* mutant germaria [24]. To determine whether the mis-expression of *hh* in escort cells contributed to the *Lsd1* mutant phenotype, we crossed both *hh*-specific UAS RNAi and *hh* mutant lines into a *c587-gal4>UAS-Lsd1^RNAi^* background. This analysis revealed that loss of *hh* function, similar to *engrailed*, suppressed the GSC-like expansion phenotype, resulting in the formation of germaria that exhibited normal germ cell differentiation (Figure 4G–J).

### Mis-expression of *engrailed* in the escort cells results in a stem cell expansion phenotype

**Figure 5:**
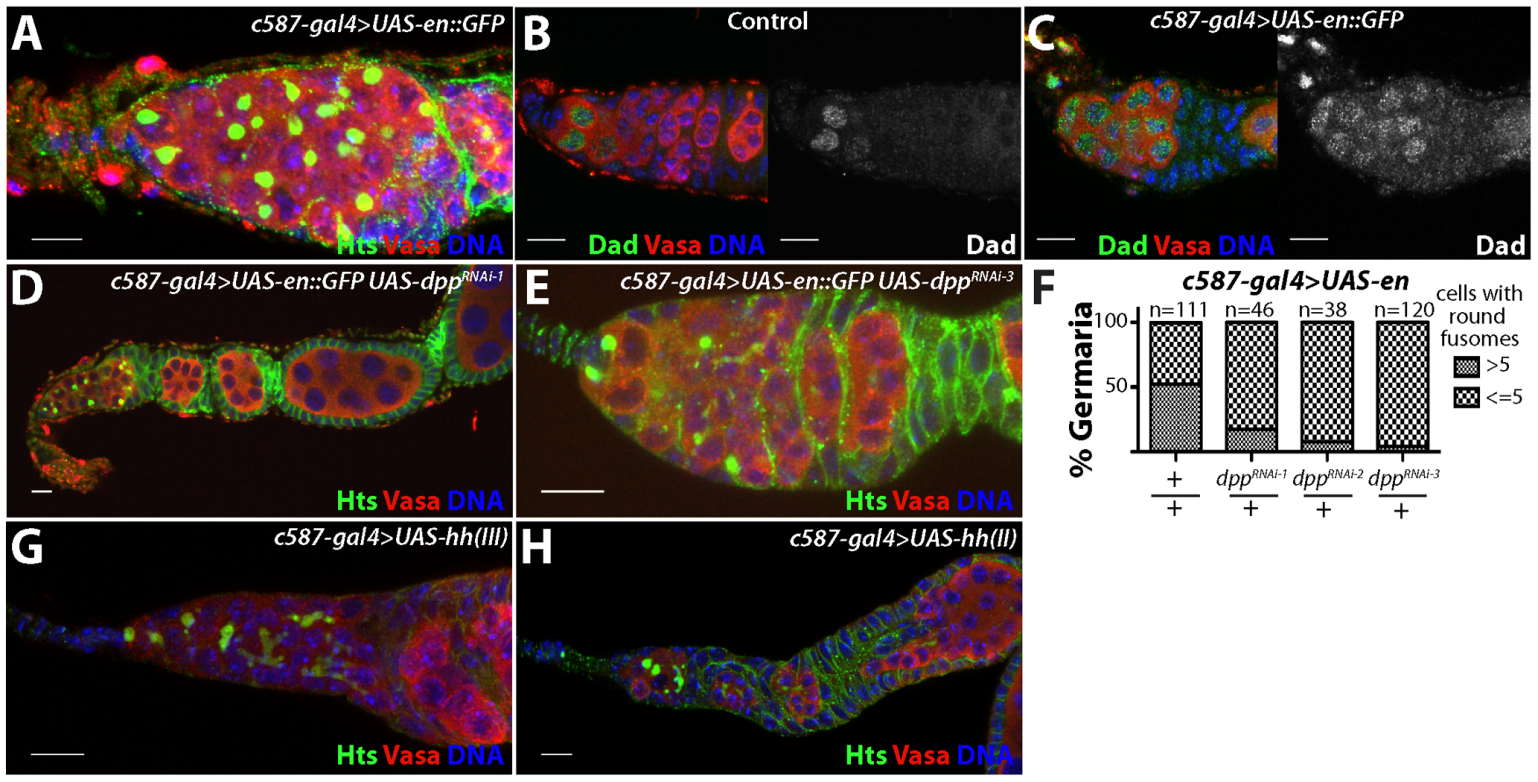
Ectopic expression of *engrailed* in the escort cells results in an expansion of stem cell-like cells in the germline. (A) *c587-gal4*>*UAS-en::GFP* germaria stained for Hts (green), VASA (red) and DNA (blue). (B) Control and (C) *c587-gal4*>*UAS-en::GFP* germaria stained for Dad-LacZ (Dad) (green), VASA (red) and DNA (blue). In control germaria, Dad-LacZ is expressed at high levels in the germline stem cells and its expression is reduced in the early differentiating cells. Germaria overexpressing *engrailed* display an expansion of Dad-LacZ positive cells. (D) Germaria from *c587-gal4*>*UAS-en::GFP*, *UAS-dpp^RNAi-1^* and (E) *c587-gal4*>*UAS-en::GFP*, *UAS-dpp^RNAi-3^* ovaries stained for Hts (green), VASA (red) and DNA (blue). Reducing the levels of *dpp* in the escort cells suppressed the *engrailed* overexpression phenotype. (F) Graph showing the percentage of germaria that contained a given number of cells with round fusomes for each genotype. (G) *c587-gal4*>*UAS-hh(III)* and (H) *c587-gal4*>*UAS-hh(II)* stained for Hts (green), VASA (red) and DNA (blue). Scale bars = 10 µM.

To assess whether mis-expression of *engrailed* and *hh* are sufficient to expand the number of stem cell-like cells in the germarium, we expressed transgenes corresponding to both genes within escort cells and early follicle cells using the *c587-gal4* driver. Similar to the phenotype exhibited by *Lsd1* mutants, ectopic expression of *engrailed* resulted in a stem cell-like cell expansion within 49.5% of germaria examined (n = 111). Many of these germline cells remained as single cells with round fusomes (Figure 5A). However, mis-expression of *engrailed* did not completely block cyst development and many ovarioles from *c587-gal4*>UAS-*engrailed* females contained maturing egg chambers.

In contrast to *engrailed*, over-expression of *hh* using two different transgenes did not obviously perturb early germ cell differentiation (Figure 5G,H). However, the mis-expression of these transgenes did result in follicle cell defects, consistent with previously published results [38]. These results indicated that the *hh* transgene is active in these cells but that *hh* over-expression in the escort cells and early follicle cells is not sufficient to induce an expansion of GSC-like cells in germaria.

Loss of *Lsd1* results in expanded BMP signaling within the germline [24]. Based on the expansion of Engrailed expression in *Lsd1* mutants and the similarities between the *Lsd1* mutant and *engrailed* over-expression phenotypes, we reasoned that mis-expression of *engrailed* may also induce ectopic BMP signaling in the ovary. To test this, we used a Dad-lacZ enhancer trap as a positive transcriptional reporter of *dpp* signal transduction [9], [39]. In control ovarioles, stem cells express high levels of Dad-LacZ, whereas the expression of this reporter sharply decreases in differentiating cysts (Figure 5B). Upon *engrailed* mis-expression in the escort cells, the number of Dad-LacZ positive germline cells increases, likely reflecting expanded Dpp signaling (Figure 5C). Next, we knocked down the expression of *dpp* in the presence of the *engrailed* transgene and found that disruption of *dpp* suppressed the *engrailed* over-expression phenotype (Figure 5D–F). Together these results suggest that mis-expression of *engrailed* in *Lsd1* mutants drives ectopic BMP signaling, resulting in the expanded GSC-like cell tumor phenotype.

**Figure 6:**
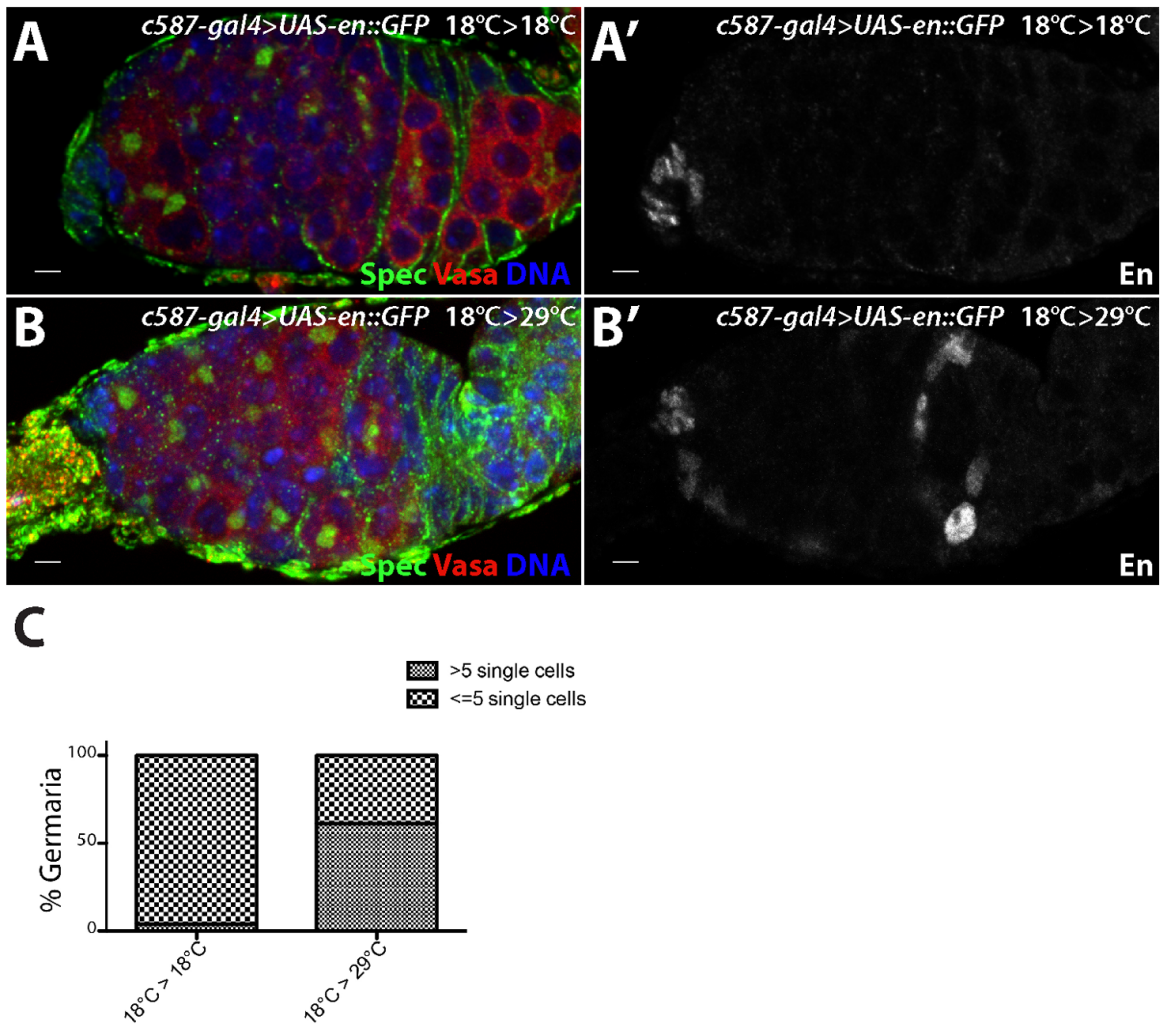
*engrailed* mis-expression in adult escort cells results in delayed germ cell differentiation. *c587-gal4*>*UAS-en::GFP* females were kept at (A) 18°C during both development and in adulthood or (B) moved to 29°C after eclosion. (A,B) The germaria were stained for alpha Spectrin (Spec) (green), VASA (red) and DNA (blue) and (A′,B′) Engrailed (En) (grayscale). (C) Graph showing the percentage of germaria with the indicated number of single germ cells with round fusomes when subjected to different temperatures. *c587-gal4*>*UAS-en::GFP* 18°C>18°C (n = 100), *c587-gal4*>*UAS-en::GFP* 18°C>29°C (n = 80). Scale bars = 10 µM.

To test whether ectopic *engrailed* expression can specifically affect adult escort cells, we performed a temporally controlled over-expression experiment. *c587-gal4*>UAS-*engrailed* larvae were kept at low temperature to prevent robust expression of the *engrailed* transgene. Ovaries from adult females maintained at a low temperature did not display ectopic Engrailed expression or an expanded undifferentiated cell phenotype (Figure 6A,A′). However, shifting *c587-gal4*>UAS-*engrailed* females to a higher temperature after eclosion resulted in ectopic *engrailed* expression in escort cells and early follicle cells, and a concomitant expansion of germline stem cell-like cells (Figure 6B–C). Thus, *engrailed* expression specifically in adults appears sufficient to induce ectopic BMP signaling in the anterior region of the germarium.

### Other Lsd1 target genes

**Figure 7:**
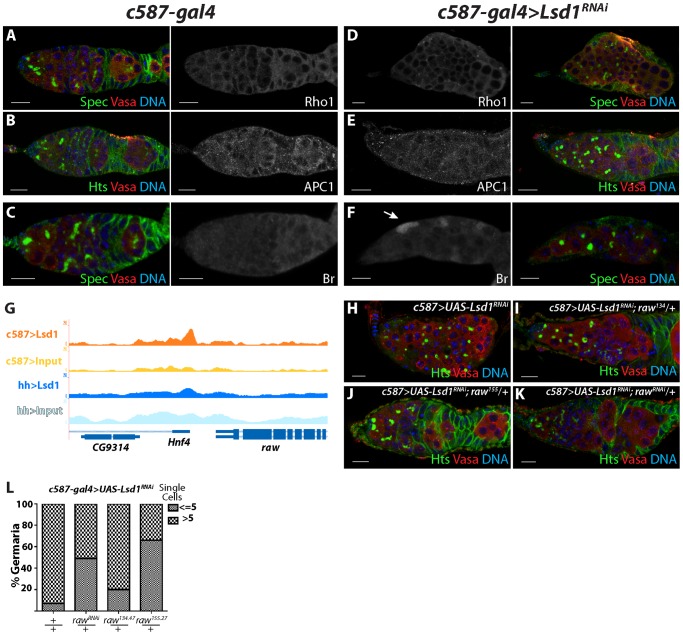
*raw* represents another potential functional Lsd1 target gene. (A–C) *c587-gal4* and (D–F) *c587-gal4*>*UAS-Lsd1^RNAi^* germaria stained for Rho1 (A,D), APC1 (B,E), and Broad (Br) (C,F). Alpha-Spectrin (Spec) or Hts (green), Vasa (red) and DNA (blue) staining of the same germaria are shown in color. (G) Screen shot of *raw* locus showing a peak of Lsd1 binding in the *c587-gal4>UASt-HA-Lsd1* sample relative to the input DNA. (H) *c587-gal4*>*UAS-Lsd1^RNAi^*, (I) *c587-gal4*>*UAS-Lsd1^RNAi^; UAS raw^135^/+* (J) *c587-gal4*>*UAS-Lsd1^RNAi^; raw^155^/+* and (K) *c587-gal4*>*UAS-Lsd1^RNAi^; raw* ^RNAi^ */+* stained for Hts (green), VASA (red) and DNA (blue). (L) Graph showing the percentage of germaria that contain the indicated number of single germ cells with round fusomes for each genotype. c*587-gal4*>*Lsd1^RNAi^* (n = 351); *c587-gal4*>*UAS-Lsd1^RNAi^; UAS raw* ^RNAi^ */+* (n = 155); *c587-gal4*>*UAS-Lsd1^RNAi^; raw^135.47^/+* (n = 224); and *c587-gal4*>*UAS-Lsd1^RNAi^; raw^155.27^/+* (n = 229). Scale bars = 10 µM.

Lsd1 associates with the promoters of many genes besides *engrailed*, some of which could potentially play a role in regulating escort cell function. To begin to characterize whether Lsd1 modulates the expression of other potential target genes, we stained *c587-gal4* control and *c587-gal4*>UAS-*Lsd1^RNAi^* ovaries using available antibodies. Cap cells and escort cells exhibit a shared peak of Lsd1 binding near the *Rho1* gene (Table S11, 12). Previous results showed that loss of *Rho1* results in escort cell defects and an expansion of GSC-like cells [3]. Knocking down *Lsd1* levels did not appear to result in any dramatic change in Rho1 expression within the escort cells (compare Figure 7A and 7D). Likewise, Lsd1 also exhibits enriched binding near *Apc1* (Tables S9, S12), a component of the Wnt signaling pathway. However antibody staining showed that Apc1 protein levels did not change to an appreciable degree upon knock-down of *Lsd1* (Figure 7B,E). In contrast, the product of a third potential Lsd1 target gene, Broad, exhibited increased expression in *c587-gal4*>UAS-*Lsd1RNAi* samples relative to controls (Figure 7C,F). However, loss of *broad* did not appear to suppress the *Lsd1* mutant phenotype (data not shown).

The *raw* gene functions in the developing gonad to regulate the morphology of somatic cells as they interact with primordial germ cells [40], [41]. Lsd1 exhibits enriched binding just 3′ to the *raw* transcription termination site (Figure 7G). Antibodies were not available to assay whether loss of *Lsd1* caused a change in Raw expression levels but *raw* mutant and RNAi lines partially suppressed the *Lsd1* phenotype (Figure 7H–L). The *raw^134.47^* allele weakly modified the GSC-like cell expansion phenotype exhibited by *c587-gal4>UAS-Lsd1^RNAi^* germaria, while both r*aw^RNAi^* and a single copy of *raw^155.27^* more strongly suppressed the *c587-gal4>UAS-Lsd1^RNAi^* phenotype, giving rise to a reduced number of germaria that carried more than 5 single cells with round fusomes. These genetic interactions suggest that mis-regulation of *raw* expression or activity also contributes to the *Lsd1* mutant phenotype.

**Figure 8:**
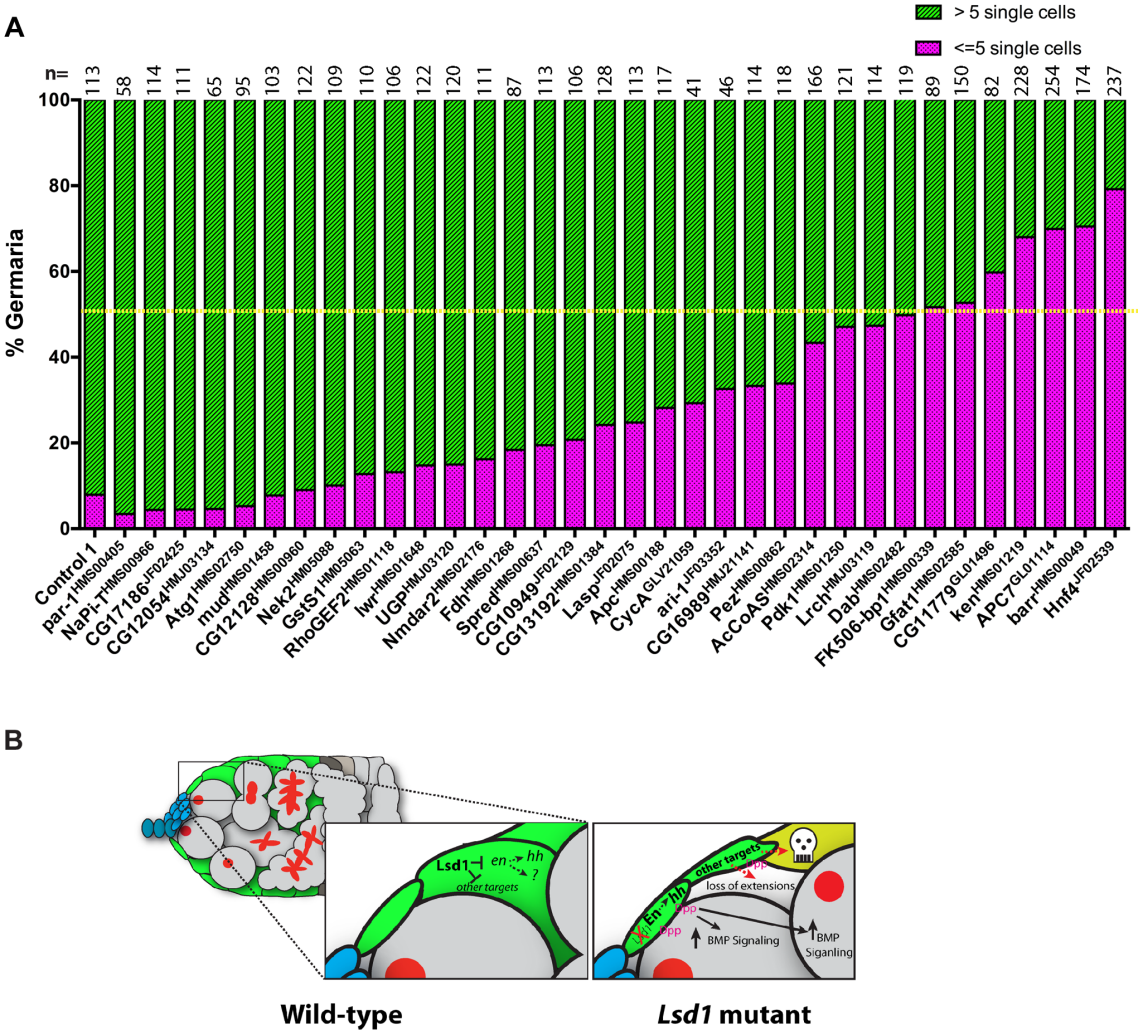
Further genetic analysis shows that reduced levels of several genes suppress the *Lsd1* mutant phenotype. (A) The graph shows the percentage of germaria that contain the indicated number of single germ cells with round fusomes for each genotype. Control 1 represents c*587-gal4*>*UAS-Lsd1^RNAi^; +/+* and the rest represent *c587-gal4*>*UAS-Lsd1^RNAi^; gene specific RNAi/+*. The resulting females were shifted to 29°C for seven days. The number (n) of germaria counted for each sample is indicated above the bar. The yellow dotted line marks the 50% threshold. All the indicated lines were crossed on their own to the *c587-gal4* driver. None of the resulting ovaries displayed phenotypes except for *CG13192^HMS01384^* (egg chamber defects) and *par-1^HMS00405^* (follicle cell defects). (B) Model of *Lsd1* function in escort cells.

Encouraged by the finding that disruption of *raw* suppressed the *Lsd1* mutant phenotype, we crossed a number of additional knockdown lines, corresponding to other putative Lsd1 target genes, into the *c587-gal4>UAS-Lsd1^RNAi^* background. We counted the total number of single germ cells with round fusomes within individual germaria from the resulting ovaries. This analysis showed that knockdown of 7 out of the 34 genes tested suppressed the *c587-gal4>UAS-Lsd1^RNAi^* phenotype to the point where fewer than 50% of the assayed germaria contained greater than 5 single cells with round fusomes (Figure 8A). This group included *FK506-binding protein 1* (*FK506-bp1)*, *Glutamine:fructose-6-phosphate aminotransferase 1*(*Gfat1*), *CG11779*, *ken and barbie* (*ken*), *Anaphase Promoting Complex subunit 7* (*APC7*), *barren* (*barr*) and *Hepatocyte nuclear factor 4* (*Hnf4*). These genes have varied functions and play roles in cell cycle regulation (*APC7* and *barr*), juvenile hormone signaling (*FK506-bp1*), development of the genitalia (*ken*) and lipid metabolism (*Hnf4*). Lack of a clear functional link between these suppressors suggests that escort cells are particularly sensitive to perturbations in their gene expression programs. Together these data show that disruption of *Lsd1* results in a complex phenotype, marked by increased BMP signaling in the germline and disruption of normal escort cell function, which likely involves the mis-expression of several direct and potentially indirect target genes (Figure 8B).

## Discussion

Using ChIP-seq, we have systematically identified Lsd1 binding sites throughout the genomes of the cap cells/terminal filament cells and the escort cells/early follicle cells of the *Drosophila* ovary. The establishment of ChIP-based techniques using these specific cell populations and this comprehensive Lsd1 genome-wide dataset will facilitate further studies on the transcriptional hierarchies that regulate escort cell and cap cell function. Although *Lsd1* mutants exhibit increased *dpp* expression [23], Lsd1 does not appear to directly target the *dpp* locus for silencing. Rather, Lsd1 represses the expression of the transcription factor *engrailed*. Mutations in the *engrailed* gene suppress the ovarian *Lsd1* mutant phenotype and mis-expression of *engrailed* in escort cells results in increased BMP signaling and an expansion of undifferentiated germline cells within germaria. Thus *engrailed* may regulate *dpp* expression in germaria through either *hh*-dependent or independent mechanisms. Lsd1 also regulates additional genes adjacent to its other binding sites. Genetic analysis shows that the mis-regulation of these other target genes likely contributes to the *Lsd1* mutant phenotype to varying degrees.

### Lsd1 gene targets and function

We have found Lsd1 associates with a limited number of loci within two different cell populations. Lsd1 exhibits fairly broad peaks of binding, ranging in size from 166–262 bp based on the FindPeaks algorithm. MACs analysis calls even wider peaks (Supplementary Tables S5, S6, S7, S8). The significance of the width of these peaks remains unclear but suggests that Lsd1 either does not associate with single sequence specific elements at these sites or exhibits a certain degree of spreading upon recruitment to a particular locus. In *Drosophila* escort cells, Lsd1 binds to over 100 sites spread throughout the genome. Lsd1 binds to fewer sites in cap cells. While some Lsd1 binding sites overlap in cap cells and escort cells, the relatively large number of different sites suggests that Lsd1 recruitment depends on multiple and perhaps distinct cell-specific co-factors. MEME analysis [42], [43] reveals that many of the identified Lsd1 binding sites contain ACTGGAA elements. GGAA sequences are often present in the core binding sites of ETS transcription factors. The *Drosophila* genome encodes a number of ETS family members, none of which have been characterized in the somatic cells of the germarium. Determining the functional relevance of these specific GGAA sites within gene promoters and identifying the transcription factors that bind to them will require additional efforts.

For technical reasons and to enable comparisons of Lsd1 binding between escort cells/early follicle cells and cap cells/terminal filament cells, we elected to express the Lsd1 HA-tagged transgenes in an otherwise wild-type background. We acknowledge the possibility that endogenous Lsd1 may outcompete the HA-tagged transgenes for binding at specific sites in the escort cells and early follicle cells. Therefore sites identified in this study may be an underrepresentation of the total number of sites bound by endogenous Lsd1. Repeating the ChIP-seq analysis using material from rescued *Lsd1^ΔN^* females that express the HA-tagged Lsd1 transgene in escort cells and early follicle cells represents important work for the future.

We found that *Lsd1* mutant samples exhibit a 6-fold increase in *engrailed* transcript levels relative to controls. Curiously, ectopic Engrailed protein expression was only observed in a small number of escort cells. Perhaps additional post-transcription mechanisms regulate the translation of Engrailed, and potentially other proteins, inside and outside of the cap cell niche. Such mechanisms would allow these cells to fine-tune their signaling output more than what could be achieved through transcriptional based mechanisms alone. Results presented here also suggest that escort cells are not uniform in nature and perhaps have specific functions or capabilities depending on their lineage and where they reside within the germarium. MARCM analysis shows that the loss of *Lsd1* in some but not all escort cells results in a marked expansion of GSC-like cells within the germarium. Previous studies have also suggested that specific escort cells have distinct roles in supporting GSCs [12].

Technical considerations aside, the severity of the *Lsd1* null phenotype compared to the *engrailed* over-expression phenotype, both in terms of the penetrance and extent of the GSC expansion phenotype and the accompanying germline and somatic cell death, suggests that *engrailed* is not the only biologically relevant target of Lsd1 regulation in the escort cells. Based on expression analysis (Figure 7), Lsd1 regulates the expression of some but not all genes adjacent to its binding sites. Our genetic analysis suggests that mis-regulation of the putative target *raw* (Figure 7) and several additional genes (Figure 8) also contribute to the *Lsd1* mutant phenotype. Characterizing the transcriptional profile of escort cells from wild-type and *Lsd1* mutant samples, which will have to await for improvements in current cell isolation and RNA profiling techniques, will help to further resolve which genes are direct and indirect targets of *Lsd1* regulation. Such approaches may also reveal additional genes that participate in niche formation and function. Of note, the observation that functionally diverse genes can suppress the *Lsd1* mutant phenotype suggests that escort cells are acutely sensitive to changes in their gene expression programs. While our data support a model that loss of *Lsd1* initially results in mis-regulation of *engrailed* and other genes that, in turn, drive GSC expansion, it is clear that many of the escort cells that experience reduced Lsd1 function retract their cellular extensions and undergo cell death. This loss of escort cells further exacerbates the GSC expansion phenotype. Given the phenotypic complexity described here and elsewhere [3], care should be taken when analyzing gene function within the escort cell population.

### Niche plasticity

How niches maintain stem cells and adjust their signaling output to ensure tissue homeostasis remains a fundamental question in stem cell biology. Elegant work has shown that terminal filament cells, cap cells and escort cells help to support the self-renewal of two to three germline stem cells at the tip of *Drosophila* germaria [5], [44]. The predominant signal emanating from the anterior tip of the germarium is Dpp, which acts locally to induce a canonical signal transduction cascade in GSCs, which in turn represses their differentiation [5], [9]. Several expression and genetic studies strongly suggest that terminal filament and cap cells, and perhaps the most anterior escort cells, are the primary source Dpp ligand [5], [9]. More recent work has suggested that Engrailed expression in cap cells non-autonomously promotes *dpp* expression in escort cells through a *hedgehog* dependent mechanism [12]. Loss of *Lsd1* results in ectopic expansion of *hh* expression in escort cells [24] and data shown here (Figure 4) reveals that disruption of *hh* partially suppresses the *Lsd1* mutant phenotype. However, consistent with previous results [38], over-expression of *hh* in escort cells does not result in an *Lsd1*-like mutant tumor phenotype (Figure 5G,H), demonstrating that *hh* is not sufficient to induce ectopic BMP signaling in the germarium. Given these observations, ectopic *engrailed* expression in escort cells likely targets additional genes besides *hh* to induce ectopic BMP signaling and promote the expansion of undifferentiated germ cells.

The finding that loss of *Lsd1* or mis-expression of *engrailed* in adult escort cells leads to expanded Dpp signal transduction within germ cells throughout the anterior portion of the germarium indicates that subpopulations of escort cells are capable, and perhaps even poised, to express *dpp* under certain conditions. Such plasticity might allow the niche to expand and contract in response to various stimuli and environmental cues. Indeed, previous studies have shown that Jak/Stat and insulin signaling can influence the number of GSCs in the ovary [45]–[49]. Moreover, ongoing dynamic regulation of signaling may be a regular feature of niches under resting homeostatic conditions. The observation that long-term knock-down of *dpp* in escort cells results in a reduced number of GSCs at the tip of the germarium, but not their complete elimination, is consistent with the notion that escort cells contribute to the maintenance of GSCs in some manner [12]. Further work, with single-cell spatial and small-scale temporal resolution, will be needed to help clarify what cells express niche signals and when.

Inappropriate and extensive expansion of niches would be predicted to upset tissue homeostasis and perhaps even result in pathological conditions. Therefore robust but flexible mechanisms that depend on chromatin factors such as Lsd1 may be in place to precisely control the expansion and contraction of *in vivo* stem cell niches. The continued study of *Drosophila* cap cells and escort cells will provide further insights into how chromatin programming regulates niche plasticity.

## Materials and Methods

### *Drosophila* stocks

*Drosophila* stocks were maintained at room temperature on standard cornmeal-agar medium unless specified otherwise. The following fly strains were used in this study: *w^1118^* was used as a control; *Lsd1^ΔN^* was provided by N. Dyson (Massachusetts General Hospital Cancer Center, Charlestown, MA); *hh-gal4* and UAS-*hh* lines [50] were provided by J. Jiang (University of Texas Southwestern, Dallas, TX); *c587-gal4* and *Dad-LacZ* were provided by A. Spradling (Carnegie Institution for Science, Baltimore, MD); the *UAS-engrailed::GFP* transgenic line was provided by Florence Maschat (Institute of Human Genetics, France); the *raw^134.47^* and *raw^155.27^* alleles were provided by Jennifer Mierisch (Loyola University of Chicago); *en^7^*, *en^4^*, *en^spt^*, *UAS-mCD8::GFP*, *UAS-dpp^RNAi-1^* (BL#-31172), *UAS-dpp^RNAi-2^* (BL#-31530), *UAS-dpp^RNAi-3^* (BL#-31531) and *UAS-raw^RNAi^* (BL#-31393), *UAS-en^RNAi-1^* (BL#-33715), *UAS-en^RNAi-2^* (BL#-26752), *UAS-hh^RNAi^* (BL#-31042), *hh^AC^* (BL#-1749), *hh^2^* (BL#-3376), *broad^npr-3^* (BL#-29971), *broad^5^* (BL#-29972), *par-1^HMS00405^* (BL#- 32410), *NaPi-T^HMS00966^* (BL#- 34003), *CG17186^JF02425^* (BL#- 27079), *CG12054^HMJ03134^* (BL#- 50910), *Atg1^HMS02750^* (BL#- 44034), *mud^HMS01458^* (BL#- 35044), *CG12128^HMS00960^* (BL#- 33997), *Nek2^HM05088^* (BL#- 28600), *GstS1^HM05063^* (BL#- 28885), *RhoGEF2^HMS01118^* (BL#- 34643), *lwr^HMS01648^* (BL#- 37506), *UGP^HMJ03120^* (BL#- 50902), *Nmdar2^HMS02176^* (BL#- 40928), *Fdh^HMS01268^* (BL#- 34937), *Spred^HMS00637^* (BL#- 32852), *CG10949^JF02129^* (BL#- 26231), *CG13192^HMS01384^* (BL#- 34390), *Lasp^JF02075^* (BL#- 26305), *Apc^HMS00188^* (BL#- 34869), *CycA^GLV21059^* (BL#- 35694), *ari-1^JF03352^* (BL#- 29416), *CG16989^HMJ21141^* (BL#- 51017), *Pez^HMS00862^* (BL#- 33919), *AcCoAS^HMS02314^* (BL#- 41917), *Pdk1^HMS01250^* (BL#- 34936), *Lrch^HMJ03119^* (BL#- 50901), *Dab^HMS02482^* (BL#- 42646), *FK506-bp1^HMS00339^* (BL#- 32348), *Gfat1^HMS02585^* (BL#- 42892), *CG11779^GL01496^* (BL#- 43155), *ken^HMS01219^* (BL#- 34739), *APC7^GL01114^* (BL#- 38932), *barr^HMS00049^* (BL#- 34068) and *Hnf4^JF02539^* (BL#- 29375) lines were obtained from the Bloomington Stock Center. *UAS-Lsd1^RNAi^* was obtained from the National Institute of Genetics, Japan.

### MARCM clones

*Lsd1* mutant MARCM clones were generated by crossing *Lsd1^ΔN^ FRT 2A* to *yw^122^ tub-gal4 UAS-GFP;;tub-gal80 FRT 2A/TM6B* (gift from Ben Ohlstein). The resulting larvae were heat-shocked twice a day at 37°C on days 5–7 after the cross was set. The resulting adult females were dissected and stained 7 days after they eclosed.

### Cloning of tagged transgenes

The HA tagged transgenes of *Lsd1* were created using Gateway Cloning (Invitrogen). The open reading frame (ORF) of *Lsd1* was cloned into modified pTHW and pPHW destination vectors (http://emb.carnegiescience.edu/labs/murphy/Gateway%20vectors.html) that contained φC31 attB sites [51]–[53]. These constructs were injected into flies and transformed using φC31 integrase into the 51D landing site on the 2^nd^ chromosome.

### Immunostaining

Adult ovaries were dissected in Grace's medium and fixed in 4% (vol/vol) formaldehyde for 10 min. The ovaries were washed with PBT (1X PBS, 0.5% BSA, and 0.3% Triton-X 100) and stained with primary antibody overnight at 4°C. The ovaries were washed and incubated in secondary antibody at room temperature for 5 hrs. Ovaries were then washed again and mounted in Vectashield containing DAPI (Vector Laboratories). The following primary antibodies were used: mouse anti-Hts (1B1) (1∶20), mouse anti-Engrailed (4D9) (1∶2), mouse anti-Broad-core (25E9.D7) (1∶10), mouse anti-Rho1 (P1D9) (1∶50) and rat anti-VASA (1∶20) (Developmental Studies Hybridoma Bank), mouse anti-β-galactosidase (1∶200) (Promega), rabbit anti-APC1 [54] (1∶1000)(gift of E. Wieschaus), Rabbit anti-α-Spectrin [55](1∶1000)(gift from Ron Dubreuil), rabbit anti-GFP (1∶1000) (Molecular Probes), rat anti-HA 3F10 (Roche) and rabbit anti-cleaved Caspase-3 (1∶250) (Cell Signaling Technology). Fluorescence-conjugated secondary antibodies (Jackson Laboratories) were used at a dilution of 1∶200.

### RNA isolation and RT-PCR

RNA was isolated from *bamΔ86* and *Lsd1^ΔN^ bamΔ86* mutant ovaries using TRIzol (Invitrogen). The RNA was treated with DNase and subjected to RT-qPCR reaction using the Superscript III First-Strand Synthesis SuperMix (Invitrogen). The primers used to amplify engrailed mRNA are as follows:

*engrailed* forward: 5′ - GCCCGCCTGGGTGTACTG

*engrailed* reverse: 5′ - CGCTTCTCGTCGTTGGTCTTG

### Chromatin immunoprecipitation and sequencing (ChIP-seq)

We used 1000 pairs of ovaries for each ChIP-seq reaction. Every 200 pairs of ovaries were dissected, fixed, washed and frozen immediately at −80°C. The entire protocol was done at 4°C unless otherwise indicated. The ovaries were dissected and fixed in 1 ml of 1% formaldehyde at room temperature for 10 mins. The crosslinking was stopped by the addition of 100 ml 1.25M glycine solution. The ovaries were washed three times with 1X cold PBS buffer and then sonicated in 500 µl ChIP Sonication Buffer (1%Triton X-100, 0.1% Deoxycholate, 50 mM Tris 8.1, 150 mM NaCl, 5 mM EDTA) on ice to achieve a final DNA length of 100 to 600 base pairs. The sample was centrifuged at maximum speed at 4°C to remove cell debris. The supernatant was transferred to a new tube and the sonicated sample was then blocked by adding Protein G agarose beads and incubating at 4°C for one hour. The beads were removed. 1% of the sample was kept aside as INPUT and to the remaining sample 3 ug rabbit-HA antibody (Abcam) was added and incubated overnight at 4°C.

The next day protein agarose G beads were added and incubated for 3 hours at 4°C. The beads was then washed well with ChIP Sonication Buffer (two times), High Salt Wash Buffer (1% Triton X-100, 0.1% Deoxycholate, 50 mM Tris 8.1, 500 mM NaCl, 5 mM EDTA) (three times), LiCl Immune Complex Wash Buffer (two times) and TE buffer. The protein bound to the beads was eluted using 500 µl Elution Buffer (1% SDS, 0.1M NaHCO_3_). The elution buffer was added to the INPUT samples and they were treated the same as the IP samples from this point. 20 µl of 5M NaCl was added to 500 µl of elution buffer and incubated at 65°C overnight.

The third day, the sample was treated with RNase A, Proteinase K and the DNA isolated using Qiagen PCR Purification kit. Subsequent library construction and sequencing of the input and immunoprecipitated DNA were conducted by the UT Southwestern McDermott Sequencing Center.

### Bioinformatic analysis of ChIP-Seq data

The primary sequencing data was mapped to the fly reference genome dm3 using BioScope (1.2.1). During the alignment, three filter steps were applied to remove low quality, ambiguous and redundant reads. HA-Lsd1 binding regions were identified as genomic regions with a significant read enrichment and binding peak profile in the ChIP reads over the input reads using the FindPeaks module in the Homer software tool [56] with 10% false discovery rate (FDR). ChIP enrichment at important genome features such as specific chromosomes, promoters, downstream, exonic, intronic and distal intergenic regions was statistically analyzed with the Cis-regulatory Element Annotation System (CEAS) [57]. *De novo* motif discovery analysis for HA-Lsd1 binding regions was performed with the Multiple EM for motif elicitation (MEME) software tool [42], [43]. High quality motifs were aligned against transcription factor motifs retrieved from JASPAR [58] and TRANSFAC [59] using the TOMTOM software tool [42] to identify known transcription factor motifs that match the MEME predicted motifs. Potential protein-coding target genes associated with the identified HA-Lsd1 binding regions were identified based on the distance of their transcription start sites (TSSs) according to their RefSeq annotation in the dm3 assembly to binding peak summits. Genes with TSSs within 5 kb or nearest to an HA-Lsd peak summit were called as target genes. ChIP-Seq data has been deposited with NCBI GEO (http://www.ncbi.nlm.nih.gov/geo) under the accession code GSE54376.

## Supporting Information

Figure S1 Expression patterns of different Lsd1 transgenes when driven by cap cell or escort cell specific drivers. All the images were captured using the same confocal settings so the relative expression levels could be compared between the samples. (A,B,C,D) Germaria stained for HA (green), Hts (red) and DNA (blue). (A′,B′,C′,D′) HA staining alone. (A) *c587-gal4>UASt-HA::Lsd1* and (B) *c587-gal4>UASp-HA::Lsd1* germaria show expression in escort cells and early follicle cells. (C) *hh-gal4>UASt-HA::Lsd1* and (D) *hh-gal4>UASp-HA::Lsd1* germaria display expression of HA::Lsd1 in the cap cells and the occasional terminal filament cell. (Scale bars, 10 µM).
(TIF)

Figure S2 The chromosomal distribution of HA::Lsd1 binding sites identified using FindPeaks in *c587-gal4>UASt-HA::Lsd1*, *c587-gal4>UASp-HA::Lsd1*, *hh-gal4>UASt-HA::Lsd1* and *hh-gal4>UASp-HA::Lsd1* germaria. The blue bars represent the percent of the genome comprised by each chromosome feature while the red bars indicate percentage distribution of Lsd1 binding sites across each chromosome feature. This analysis reveals that, while hh>pTHW displays a modest enrichment for binding on the right arm of chromosome 3, in general, Lsd1 binding appears evenly distributed across the genome.
(TIF)

Figure S3 The distribution of HA::Lsd1 binding sites relative to gene features in *c587-gal4>UASt-HA::Lsd1*, *c587-gal4>UASp-HA::Lsd1*, *hh-gal4>UASt-HA::Lsd1* and *hh-gal4>UASp-HA::Lsd1* samples.
(TIF)

Figure S4 Read depth plots for input DNA, H3K4me3 and H3K4me1 ChIP DNA from the modENCODE project within +/−3 kb of superimposition of all Lsd1 binding sites (defined as 0). Valleys of H3K4me levels exist in regions corresponding to escort cell Lsd1 binding sites.
(TIF)

Figure S5 (A) Gene ontology hierarchies of the next gene adjacent to escort cell specific Lsd1 binding sites (no distance cutoff) or those genes with transcriptional start sites within 2.5 kb of Lsd1 binding sites based on the UASt-HA::Lsd1 data sets [28], [29]. Yellow to Orange represents less significant to more significant terms. The size of nodes corresponds to the number of genes in the query set that belong to the category. (B) A motif enriched in Lsd1 binding sites detected by MEME analysis.
(TIF)

Figure S6 (A) *bam^Δ86^* and (B) *bam^Δ86^ Lsd1^ΔN^* double mutant germaria stained for Hts (green), Vasa (red) and DNA (blue). Both the single and double mutant germaria are roughly the same size and comprised of similar cell types. Scale bars = 10 µM.
(TIF)

Table S1 FindPeaks output for *c587-gal4>UASp-HA::Lsd1* ChIP-seq.
(XLSX)

Table S2 FindPeaks output for *c587-gal4>UASt-HA::Lsd1* ChIP-seq.
(XLSX)

Table S3 FindPeaks output for *hh-gal4>UASp-HA::Lsd1* ChIP-seq.
(XLSX)

Table S4 FindPeaks output for *hh-gal4>UASt-HA::Lsd1* ChIP-seq.
(XLSX)

Table S5 MACs output for *c587-gal4>UASp-HA::Lsd1* ChIP-seq.
(XLS)

Table S6 MACs output for *c587-gal4>UASt-HA::Lsd1* ChIP-seq.
(XLS)

Table S7 MACs output for *hh-gal4>UASp-HA::Lsd1* ChIP-seq.
(XLS)

Table S8 MACs output for *hh-gal4>UASt-HA::Lsd1* ChIP-seq.
(XLS)

Table S9 Genes near escort cell and early follicle cell peaks.
(XLSX)

Table S10 Genes near cap cells and terminal filament cells.
(XLSX)

Table S11 Genes near shared peaks.
(XLSX)

Table S12 Genes within 5 kb of UASt-HA::Lsd1 binding peaks.
(XLSX)
