# Supplementary figures and images for: Lsd1 Restricts the Number of Germline Stem Cells by Regulating Multiple Targets in Escort Cells
Source: PLoS Genet. 2014 Mar 13;10(3):e1004200. doi: 10.1371/journal.pgen.1004200 (PMC3952827; doi:10.1371/journal.pgen.1004200)

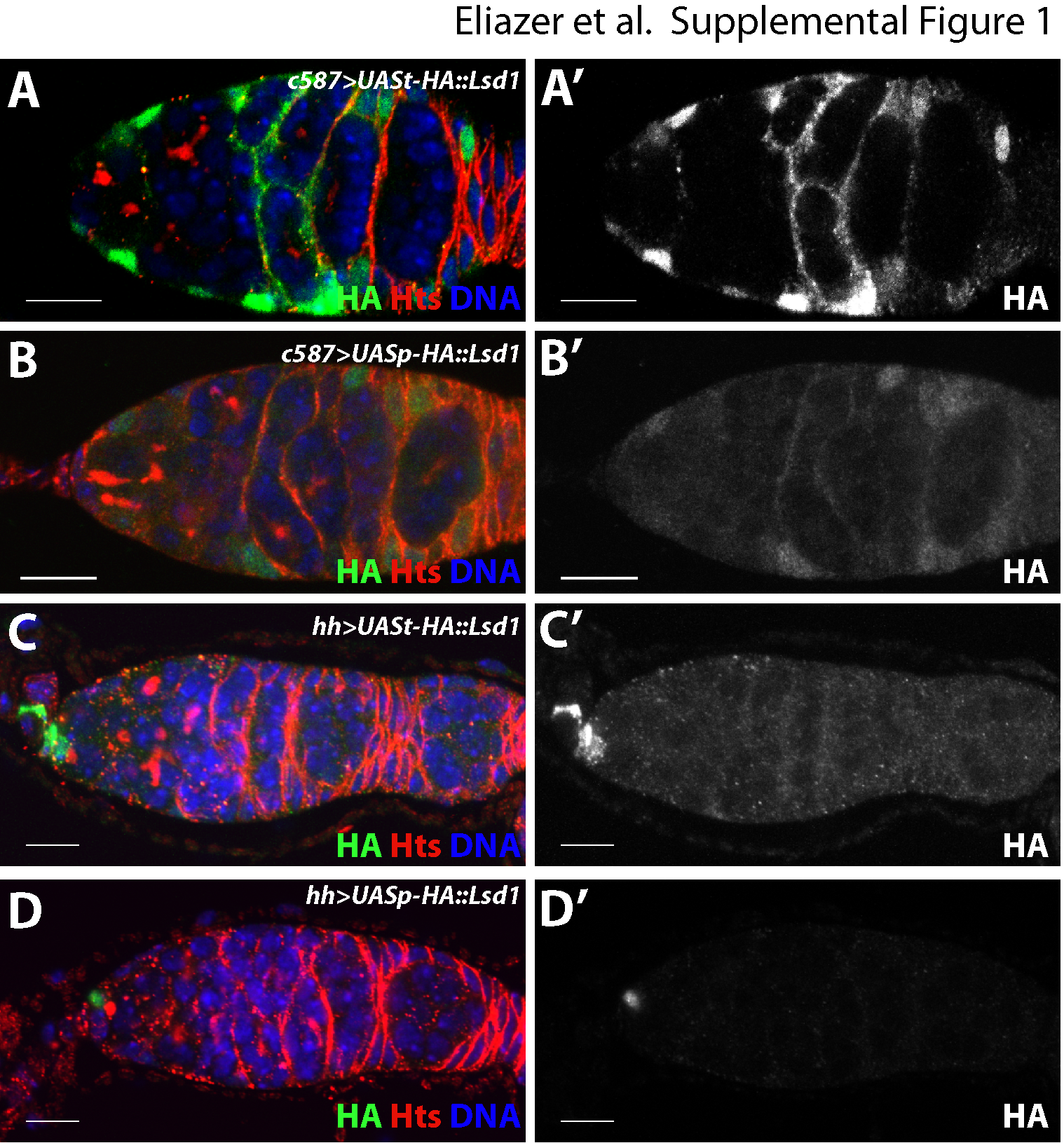

Supplement: Figure S1 — Expression patterns of different Lsd1 transgenes when driven by cap cell or escort cell specific drivers. All the images were captured using the same confocal settings so the relative expression levels could be compared between the samples. (A,B,C,D) Germaria stained for HA (green), Hts (red) and DNA (blue). (A′,B′,C′,D′) HA staining alone. (A) c587-gal4>UASt-HA::Lsd1 and (B) c587-gal4>UASp-HA::Lsd1 germaria show expression in escort cells and early follicle cells. (C) hh-gal4>UASt-HA::Lsd1 and (D) hh-gal4>UASp-HA::Lsd1 germaria display expression of HA::Lsd1 in the cap cells and the occasional terminal filament cell. (Scale bars, 10 µM). (TIF) [file pgen.1004200.s001.tif]

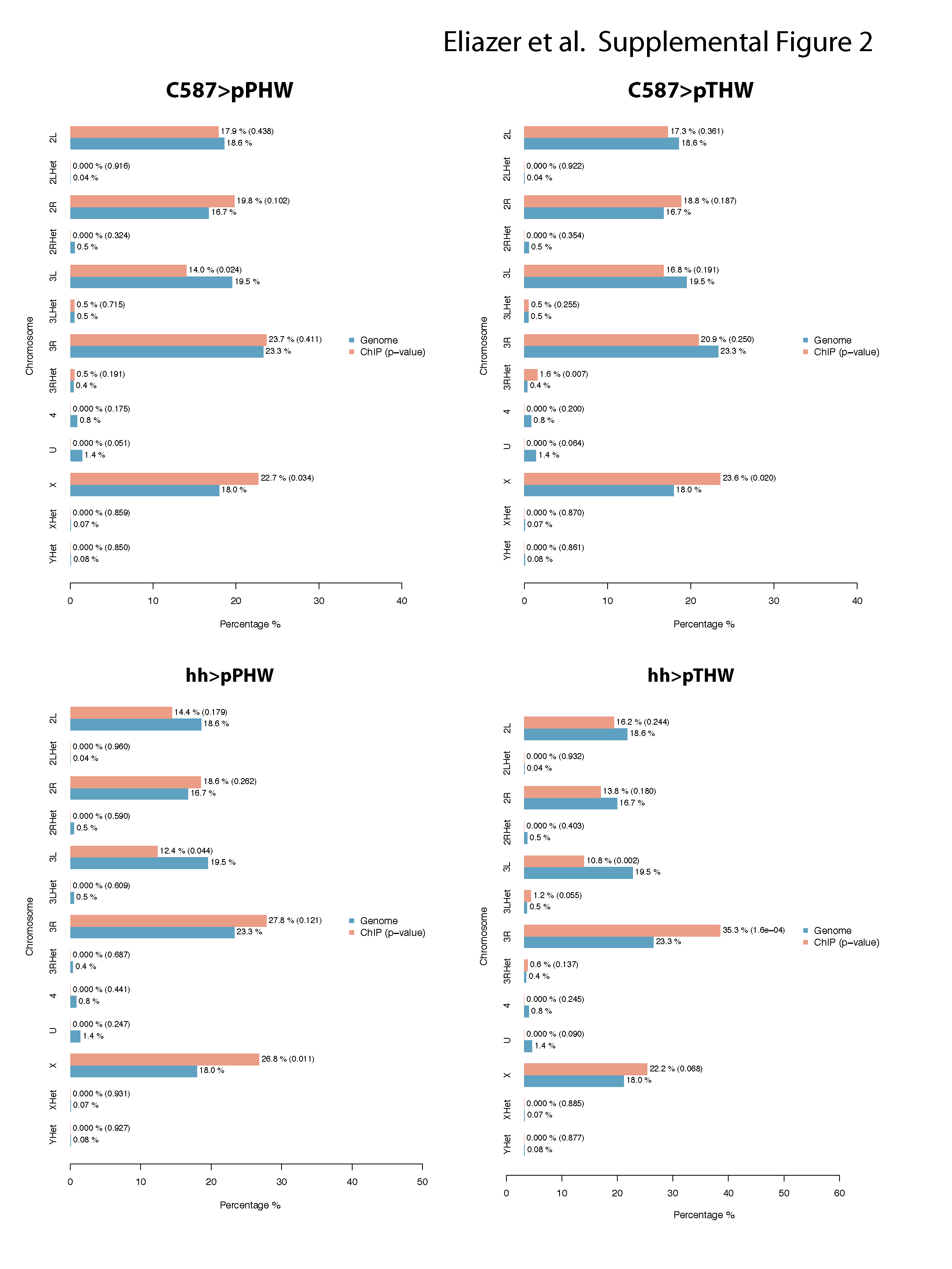

Supplement: Figure S2 — The chromosomal distribution of HA::Lsd1 binding sites identified using FindPeaks in c587-gal4>UASt-HA::Lsd1, c587-gal4>UASp-HA::Lsd1, hh-gal4>UASt-HA::Lsd1 and hh-gal4>UASp-HA::Lsd1 germaria. The blue bars represent the percent of the genome comprised by each chromosome feature while the red bars indicate percentage distribution of Lsd1 binding sites across each chromosome feature. This analysis reveals that, while hh>pTHW displays a modest enrichment for binding on the right arm of chromosome 3, in general, Lsd1 binding appears evenly distributed across the genome. (TIF) [file pgen.1004200.s002.tif]

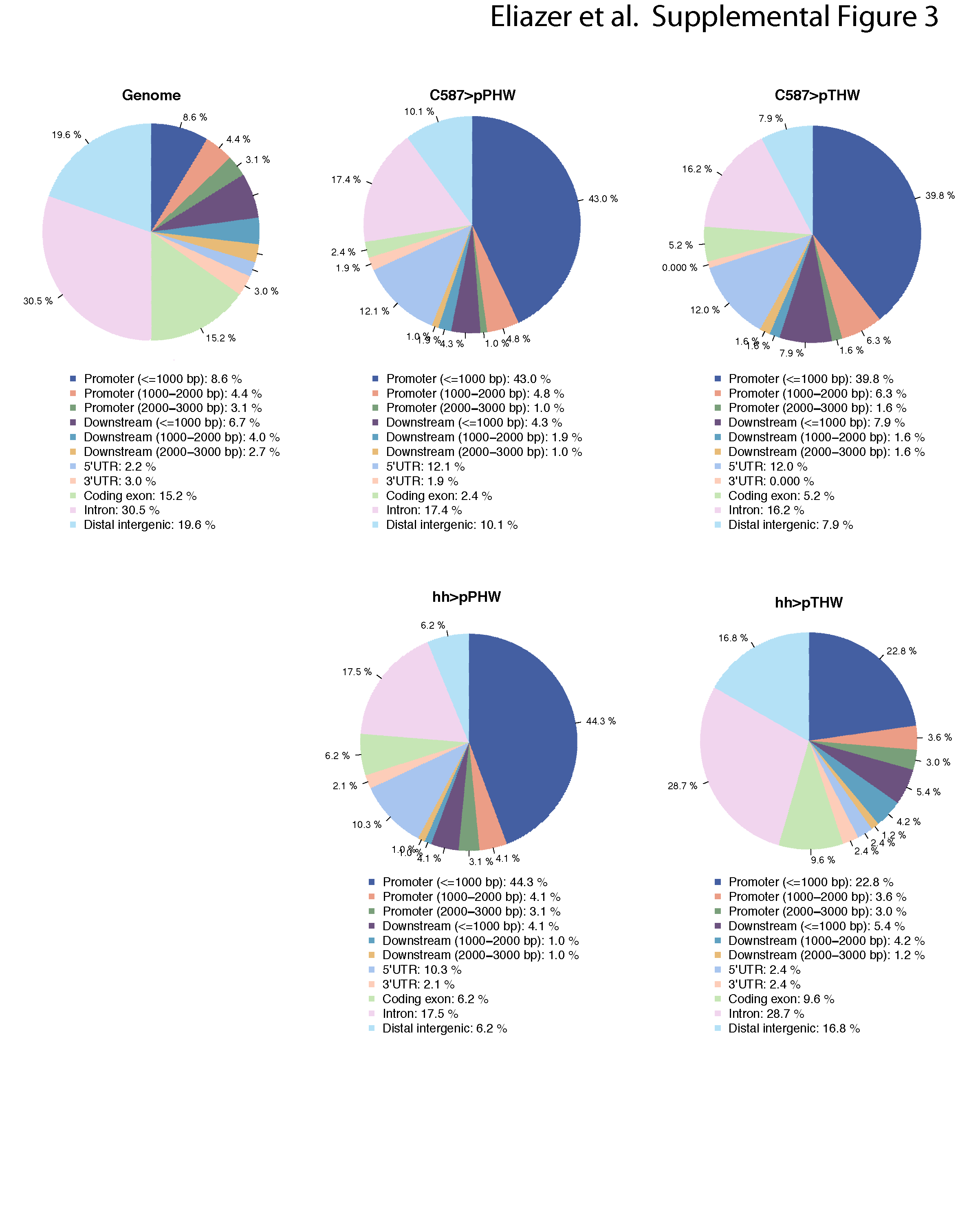

Supplement: Figure S3 — The distribution of HA::Lsd1 binding sites relative to gene features in c587-gal4>UASt-HA::Lsd1, c587-gal4>UASp-HA::Lsd1, hh-gal4>UASt-HA::Lsd1 and hh-gal4>UASp-HA::Lsd1 samples. (TIF) [file pgen.1004200.s003.tif]

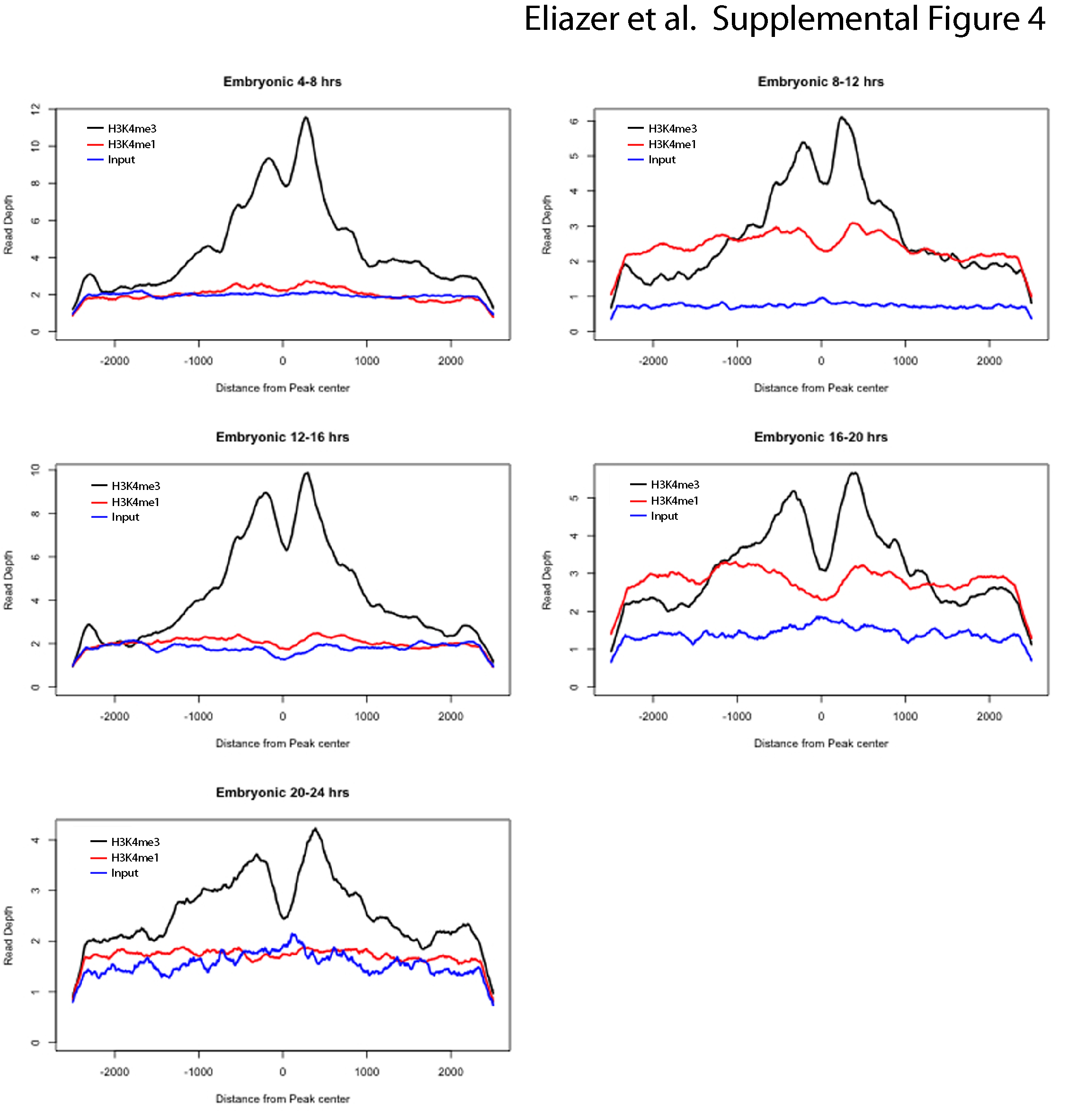

Supplement: Figure S4 — Read depth plots for input DNA, H3K4me3 and H3K4me1 ChIP DNA from the modENCODE project within +/−3 kb of superimposition of all Lsd1 binding sites (defined as 0). Valleys of H3K4me levels exist in regions corresponding to escort cell Lsd1 binding sites. (TIF) [file pgen.1004200.s004.tif]

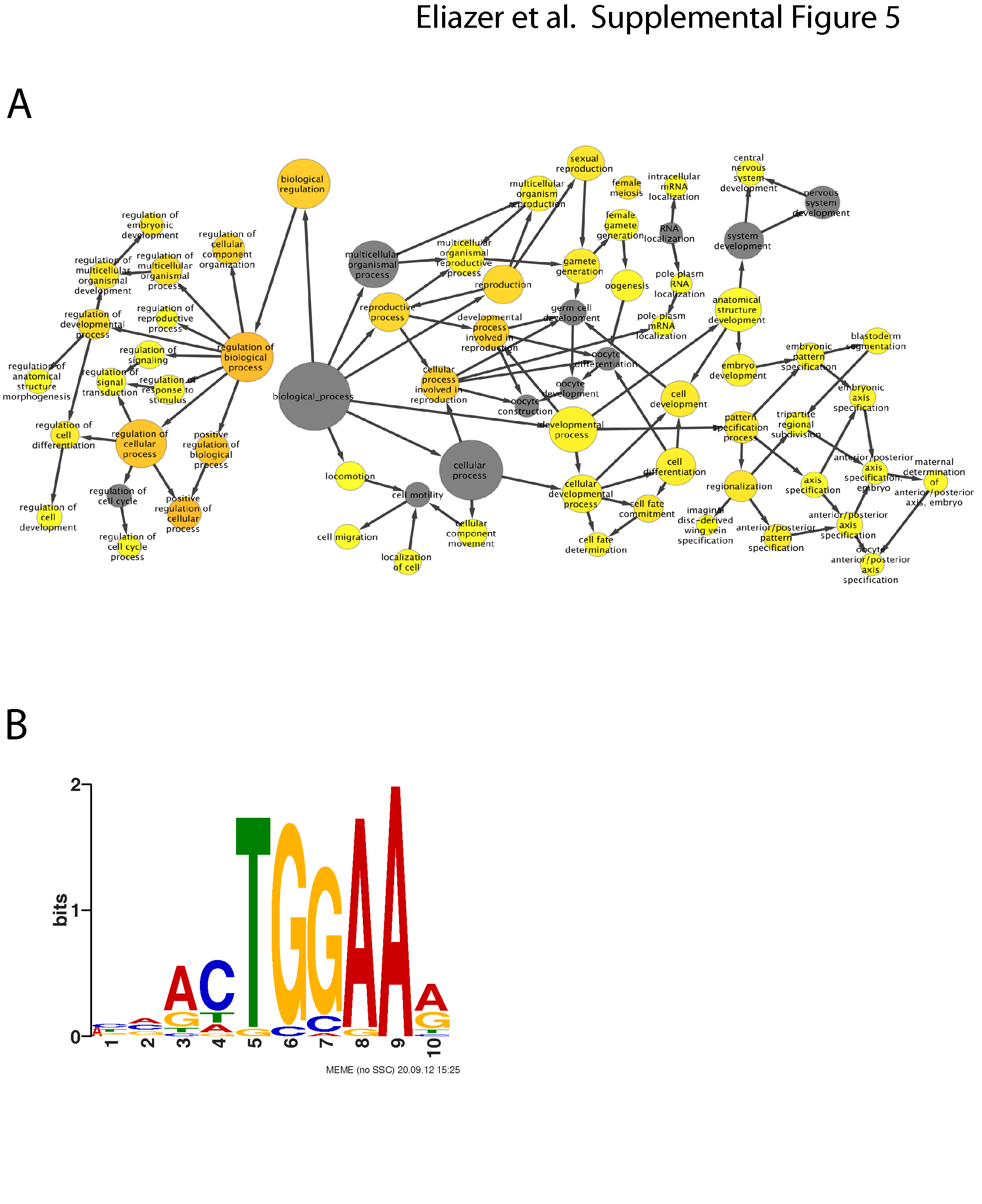

Supplement: Figure S5 — (A) Gene ontology hierarchies of the next gene adjacent to escort cell specific Lsd1 binding sites (no distance cutoff) or those genes with transcriptional start sites within 2.5 kb of Lsd1 binding sites based on the UASt-HA::Lsd1 data sets [28], [29]. Yellow to Orange represents less significant to more significant terms. The size of nodes corresponds to the number of genes in the query set that belong to the category. (B) A motif enriched in Lsd1 binding sites detected by MEME analysis. (TIF) [file pgen.1004200.s005.tif]

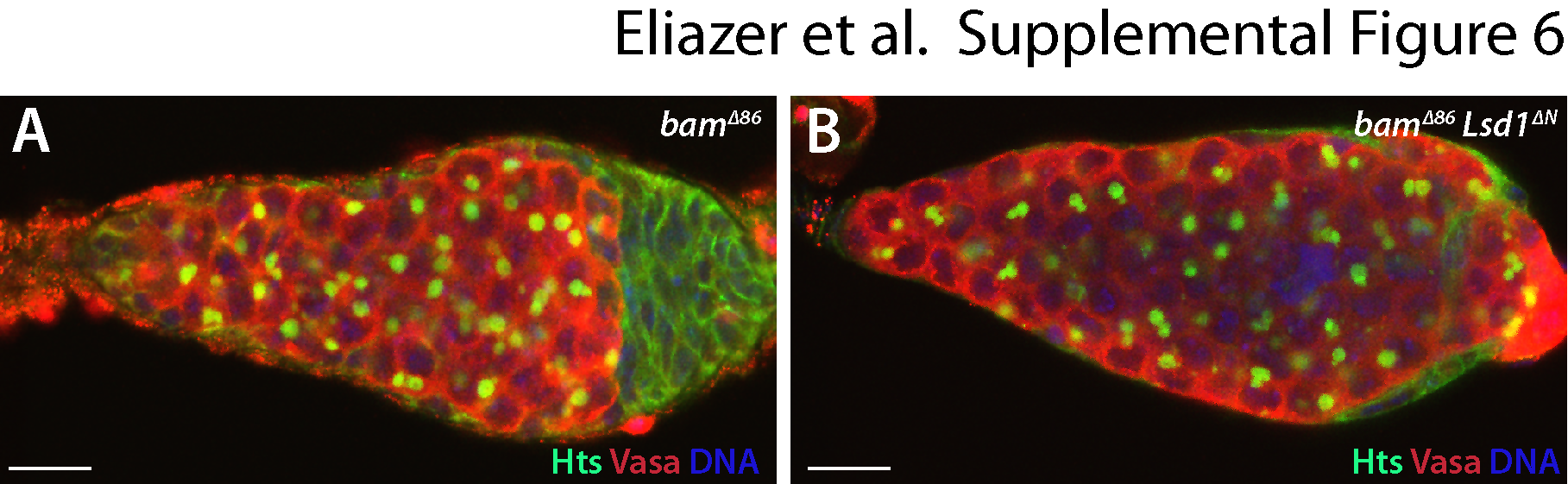

Supplement: Figure S6 — (A) bamΔ86 and (B) bamΔ86 Lsd1ΔN double mutant germaria stained for Hts (green), Vasa (red) and DNA (blue). Both the single and double mutant germaria are roughly the same size and comprised of similar cell types. Scale bars = 10 µM. (TIF) [file pgen.1004200.s006.tif]
